# Anhedonia Relates to Increased Psychomotor Retardation Using an Instrumental Handwriting-Based Measure

**DOI:** 10.1007/s10862-025-10249-1

**Published:** 2025-09-22

**Authors:** Riley E. Maher, David M. Klemballa, Sebastian Walther, Vijay A. Mittal, Stewart A. Shankman, Allison M. Letkiewicz

**Affiliations:** 1 Department of Psychiatry and Behavioral Sciences, Northwestern University, Chicago, IL USA; 2 Department of Psychology, Northwestern University, Evanston, IL USA; 3 Translational Research Center, University Hospital of Psychiatry and Psychotherapy, University of Bern, Bern, Switzerland

**Keywords:** Depression, Psychomotor slowing, Psychomotor retardation, Anhedonia, Velocity scaling, Reliability, Instrumental measure, Kinematics, Handwriting

## Abstract

Psychomotor retardation (PmR) is a clinically important but understudied feature of depression. Available studies of PmR in depression have also been limited by the reliance on self-report and observation. Instrumental measures, which have been used to assess motor symptoms in neurological disorders, exhibit greater precision and sensitivity than self-report measures and can provide vital information in the clinical presentation. Hence, the present study sought to examine (a) whether depression (diagnosis and/or symptoms) is abnormally associated with an instrumental-based indictaor of PmR, velocity scaling (VS), a behavioral metric that reflects the ability to increase rate of movement across longer versus shorter distances, and (b) the reliability (internal consistency) of VS. A large sample of healthy controls (*n* = 85) and those with lifetime MDD (*n* = 146), completed a handwriting task on a tablet. VS was quantified as a slope of change in velocity from 1 to 4 cm hand-drawn loops, with steeper slopes indicating less PmR. Whereas VS did not differ between groups, lower VS was significantly negatively related to greater anhedonia across participants, even after covarying for overall depression severity. VS scores also exhibited excellent internal consistency. The handwriting task is a reliable and brief measure that can be completed in only a few minutes and could be a feasible way to assess for PmR in clinical settings. Future studies are needed to determine the temporal relation between VS and anhedonia.

**Supplementary Information:**

The online version contains supplementary material available at 10.1007/s10862-025-10249-1.

## Introduction

Major depressive disorder (MDD) is costly, highly prevalent, and is a leading cause of disease burden worldwide. Indeed, MDD negatively impacts health, relationships, and work functioning (Hays et al., 1995; Mathers & Loncar, 2006), and approximately 20% of individuals will meet criteria for MDD in their lifetime (Hasin et al., 2018). Given the extensive psychosocial and economic impact of MDD, identifying behavioral indicators of depression is critical, work which can inform the development of more effective treatments.

MDD is very heterogeneous in its clinical presentation. A vastly understudied feature of MDD is psychomotor retardation (PmR), which is characterized as a slowing or reduction in physical movements, e.g., “talking or moving more slowly than usual” (American Psychiatric Association, 2013; Shankman et al., 2020; Sobin & Sackheim, 1997; Walther et al., 2018). PmR and other motor disturbances are (a) particularly common in more severe cases of MDD, (b) related to poorer treatment response, (c) related to familial risk for MDD, and (d) characterized by a qualitatively different pattern of neural connectivity (Shankman et al., 2020; Ulbricht et al., 2018; Wüthrich et al., 2023; Damme et al., 2022).

Studies have used a variety of methods to assess PmR, including self- and observer-report, activity monitoring, and assessments of gross body movement and motor speed (Dean & Mittal, 2015; Lohr et al., 2013; Schrijvers et al., 2008; van Harten et al., 2017; for review, see Shankman et al., 2020). The majority of these methods, however, are extremely coarse, focusing on overt indications of slowing (e.g., perceptions of whether the person’s body movement looks slower; Shankman et al., 2020). By contrast, instrumental tasks can elucidate subtler and more fine-grained presentations of PmR and other psychomotor disturbances that are not apparent with traditional measures (Wüthrich et al., 2022). Whereas instrumental measures of PmR have been used to assess psychomotor disturbance in Parkinson’s disease, psychosis spectrum disorder, autism spectrum disorder, and attention deficit-hyperactivity disorder (Caligiuri et al., 1998; Dean & Mittal, 2015; Grace et al., 2018; Koning et al., 2011; Langmaid et al., 2014; van Gemmert et al., 1998), and to assess for potential side effects of medication (e.g., antipsychotics; Caligiuri et al., 2019; Caligiuri et al., 2022), they have rarely been used in studies of MDD.

Handwriting-based kinematic measures are well-established instrumental assessment tools that can quantify subtle presentations of PmR. One particular handwriting measure, velocity scaling (VS), has been used as a novel indicator of PmR (Caliguri et al., 2006; Vessio et al., 2019). VS is the ability to modify the velocity (i.e., rate) of one’s movement (Caligiuri et al., 1998; Caliguri et al., 2006). More specifically, VS allows an individual to change their rate of movement in proportion to distance traveled - traveling across greater distances requires an individual to move at a relatively faster velocity than shorter distances (Caligiuri & Ellwanger, 2000). Slower VS has been found in individuals with Parkinson’s Disease and in patients with schizophrenia with drug-induced extrapyramidal side effects, which are dysfunctional movements that can result from dopamine-receptor blocking agents get (D’Souza & Hooten, 2018; Caliguri et al., 2006; Caligiuri et al., 2009; Cortese et al., 2005). VS performance has also been found to be slower among those at ultrahigh risk for psychosis and correlated with negative symptoms of schizophrenia, such as avolition and anhedonia (Dean & Mittal, 2015). Notably, bradykinesia and movement disorders characterized by motor slowing, including Parkinson’s Disease, show more consistent impairment in VS than in simple velocity and reaction time (Warabi et al., 1986; Pfann et al., 2001), indicating that VS is a particularly important feature of PmR.

Although handwriting-based kinematic metrics are promising measures of PmR and there is preliminary evidence of lower VS in MDD using wrist-flexion measures (Caligiuri & Ellwanger, 2000; Lohr et al., 2013; though Caligiuri et al., 2003 did not find a group effect of MDD), handwriting-based measures of VS have not been used to assess VS in MDD. Notably, handwriting tasks are more feasible for use in clinical settings than wrist flexion tasks as they require more widely available hardware (e.g., tablets and pens, and perhaps even the touchscreen of a cell phone) while wrist flexion tasks (which assess changes in wrist rotation) require a specialized joystick (Lohr et al., 2013).

The psychometric properties (e.g., validity, reliability) of handwriting-based VS have also not yet been established. The use of valid, reliable, and feasible instrumental measures of PmR is vital for accurately assessing impairment and changes in symptoms that may result from treatment in clinical settings and/or side effects of medication (Caligiuri et al., 2003; Caliguiri et al., 2009). More generally, utilizing measures with good psychometric properties is important for research on individual differences, such as motor functioning and psychiatric symptoms (Clayson, 2024; Kimberlin & Winterstein, 2008). A few studies previously examined the reliability of handwriting metrics (e.g., writing size, speed, velocity) in healthy controls, individuals with Parkinson’s Disease, Alzheimer’s Disease, and patients with bipolar disorder (Caligiuri et al., 2010; Caligiuri & Mohammed, 2019; Nackaerts et al., 2017; Nusret et al., 2022). Although these studies indicate that overall handwriting metrics exhibit good-to-excellent internal consistency for velocity (e.g., Caligiuri et al., 2010; Caligiuri & Mohammed, 2019), VS specific psychometrics have only been established for wrist flexion (Caligiuri et al., 1998; Caligiuri & Ellwanger, 2000; Cortese et al., 2005).

The primary goals of the present study were to examine (a) associations between a handwriting-based instrumental measure of PmR and depression (both the categorical diagnosis of MDD and dimensional severity measures of depression) and (b) the reliability of this instrumental measure. To capture depression dimensionally, we assessed general symptoms of depression. Additionally, anhedonia, which is a specific dimension of depression, was assessed because it is particularly pernicious dimension of depression that is related to altered basal ganglia functioning and to poorer VS performance in individuals at risk for psychosis (Dean & Mittal, 2015; Rizvi et al., 2016; Treadway & Zald, 2011; Weinberg et al., 2015). Given previous research showing instrumental assessment-based motor abnormalities (including VS) in individuals with current MDD relative to healthy controls and individuals with remitted MDD (e.g., Caligiuri et al., 2003; Lohr et al., 2013; Sabbe et al., 1999), it was hypothesized that participants with current MDD would produce lower VS scores than healthy controls and individuals with remitted MDD. It was also anticipated that, across participants, individuals with greater symptoms of depression (and particularly anhedonia) would exhibit lower VS scores than individuals with lower symptoms of depression. It was hypothesized that the handwriting task would also produce reliable velocity and VS scores, as indicated by internal consistency. Although our primary aim was to examine associations with depression and the psychometric properties for VS, we also examined these factors with velocity to identify whether depression-related effects are specific to VS. Because previous studies have shown greater/more consistent effects for VS than velocity in motor-related disorders, it was hypothesized that depression-related effects would be stronger for VS than velocity.

## Methods

### Participants

Adults (*N* = 242) were recruited from the Chicago, Illinois community from November 2019 to December 2023 for an ongoing NIMH-funded study examining psychomotor functioning in depression. Primary inclusion criteria were: (1) 18–60 years of age, (2) able to read and comprehend English, (3) able to provide informed consent, (4) right-handed, and (5) met group-specific inclusion/exclusion criteria (described below). Primary exclusion criteria were: (1) personal or first-degree family history of psychosis, mania, or hypomania, (2) head injury, (3) neurological conditions, tic disorders, or Lifetime ADHD, (4) psychotropic medications that impact motor function (e.g., continuously administered medications that affect dopaminergic functioning, such as immunomodulators and certain antidepressants, such as bupropion and nortriptyline), (5) current moderate or severe alcohol/substance use disorder, and (6) a contraindication to the MRI scanning environment (for other aspects of the study), such as a permanent metal in the body, history of metalwork, large tattoos, or current pregnancy.

Group-specific inclusion/exclusion criteria for the present study were based on diagnoses assessed using the Structured Clinical Interview for DSM-5 (SCID-5; First et al., 2015). Participants were included in one of three groups: (1) healthy control (HC) group, if they did not meet lifetime criteria for any major DSM-5 disorder, (2) remitted MDD (rMDD) group, if they met past, but not current criteria for MDD and did not have a major current DSM disorder (i.e., anxiety, trauma, substance/alcohol use disorder, obsessive-compulsive spectrum, or an eating disorder), or (3) current MDD (cMDD) group, if they met current DSM-5 criteria for MDD. A final sample of *N* = 231 participants were included in the study (HC *n* = 85; rMDD *n* = 97; cMDD *n* = 49; 11 additional participants from the larger study did not complete the Handwriting Task, e.g., due to time constraints, and thus were not included in the present study).

All procedures were approved by the Northwestern University Institutional Review Board. All participants provided written informed consent and were financially compensated. The data from the experiments reported here are available in the NIH National Data Archive.

## Assessments and Measures

### Structured Clinical Interview for DSM-5 Disorders (SCID-5)

Current and lifetime psychiatric diagnoses were assessed using the SCID-5 (First et al., 2015). Diagnosticians were trained to criterion by watching SCID-101 training videos and completing three interviews observed by an advanced interviewer with diagnoses made in full agreement with the observer. Assessments were supervised by a licensed clinical psychologist (coauthor SAS) and regular supervision meetings were dedicated to discussing individual interviews and creating consensus ratings. Psychotropic medications were also assessed during the SCID-5 interview (see Table 1).

**Table 1:** Demographics

*N* = 231		HC (*n* = 85)	rMDD (*n* = 97)	cMDD (*n* = 49)	F-value/ χ2-value
**Sex**	Female (%)	52 (61.2%)	57 (58.8%)	37 (75.5%)	χ2(2) = 4.16, *p* =.125
**Gender**	Female (%)	48 (56.5%)	50 (51.5%)	33 (67.3%)	**χ2(4) = 9.82**, ***p*** **=.044**
	Male (%)	34 (48.8%)	38 (39.2%)	12 (24.4%)	
	Gender minority (%) Gender-fluid, non-binary, etc.	4 (4.7%)	9 (9.3%)	4 (8.2%)	
**Age** *M (SD)*	Years	29.9 (9.8)	28.8 (9.4)	29.2 (10.3)	*F*(2,229) = 0.26, *p* =.768
**Ethnicity**	Hispanic/Latine (%)	13 (15.3%)	17 (17.5%)	12 (24.5%)	χ2(2) = 1.82, *p* =.404
**Race**	Asian	24 (28.2%)	12 (12.4%)	6 (12.2%)	χ2(6) = 11.82, *p* =.066
	Black/African American	11 (12.9%)	11 (11.3%)	9 (18.4%)	
	Multiracial/Unknown	13 (15.3%)	14 (14.4%)	6 (12.2%)	
	White	37 (43.5%)	60 (61.9%)	28 (57.1%)	
**IDAS** *M (SD)*	General Depression (anhedonia items removed)	24.6 (7.2)	32.1 (9.4)	52.5 (11.4)	***F*(2**,**227) = 145.78**, ***p*** **<.05**
	Well-Being^1^	24.2 (6.7)	22.5 (6.7)	16.6 (5.9)	***F*(2**,**229) = 22.30**, ***p*** **<.05**
**Medication**	SSRI	5 (5.8%)	24 (25.0%)	7 (14.3%)	^2^**χ2(2) = 19.27**, ***p*** **<.05**
	SNRI	1 (1.2%)	6 (10.4%)	4 (8.3%)	
	NDRI	1 (1.2%)	8 (8.3%)	5 (10.2%)	
	SARI	0 (0%)	4 (4.2%)	1 (2.0%)	
	Benzodiazepine	0 (0%)	0 (0.0%)	2 (4.1%)	
	Other Psychotropic	1 (1.2%)	4 (4.1%)	3 (6.1%)	
	Any Medication^2^	7 (8.2%)	34 (35.1%)	17 (34.7%)	
**# of Medications**	0	79 (91.9%)	64 (65.6%)	32 (65.3%)	***F*(2**,**229) = 9.51**, ***p*** **<.05**
	1	6 (6.7%)	22 (22.9%)	12 (24.5%)	
	2	1 (1.1%)	9 (9.4%)	4 (8.2%)	
	3+	0 (0%)	1 (1.0%)	1 (2.0%)	
^1^Invetory of Depression and Anxiety Symptoms (IDAS) Well-Being was multiplied by −1 to represent “Anhedonia” in the analyses ^2^Any Medication comparison (0 = none, 1 = 1 or more medications) SSRI = Selective Serotonin Reuptake Inhibitor, SNRI = Selective Serotonin and Norepinephrine Reuptake Inhibitor, NDRI = Norepinephrine Dopamine Reuptake Inhibitor, SARI = Serotonin Antagonist and Reuptake Inhibitor					

### Inventory of Depression and Anxiety Symptoms (IDAS-II)

The IDAS-II (Watson et al., 2012) is a 99-item questionnaire that measures current symptoms of depression and anxiety. In the current study, a subset of the IDAS-II scales was selected to capture two related, yet distinct, dimensions of depression: (1) Anhedonia (the Well-Being subscale multiplied by −1 for reverse coding, Letkiewicz et al., 2022) and (2) General Depression (with the anhedonia items removed). The IDAS-II reliabilities were in the excellent range, with Cronbach’s α’s of 0.90 and 0.94, respectively. Across all participants, Anhedonia and General Depression were moderately correlated at *r* =.55.

### Handwriting Task and Data Processing

Handwriting samples were acquired using Neuroscript MovAlyzeR software (http://www.neuroscript.net), installed on a Fujitsu T901 tablet computer, and a non-inking pen. The Handwriting Task is programmed to divide the writing surface into 1 and 4 cm regions, which are marked with horizontal lines. To ensure that there was viable data for each condition, participants were sometimes asked to redo a trial; for example, if there was a pen lift during the trial or if the participant completed the condition incorrectly (e.g., they wrote 7 loops instead of 8).

#### LL1 and LL4 Conditions

**Fig. 1:**
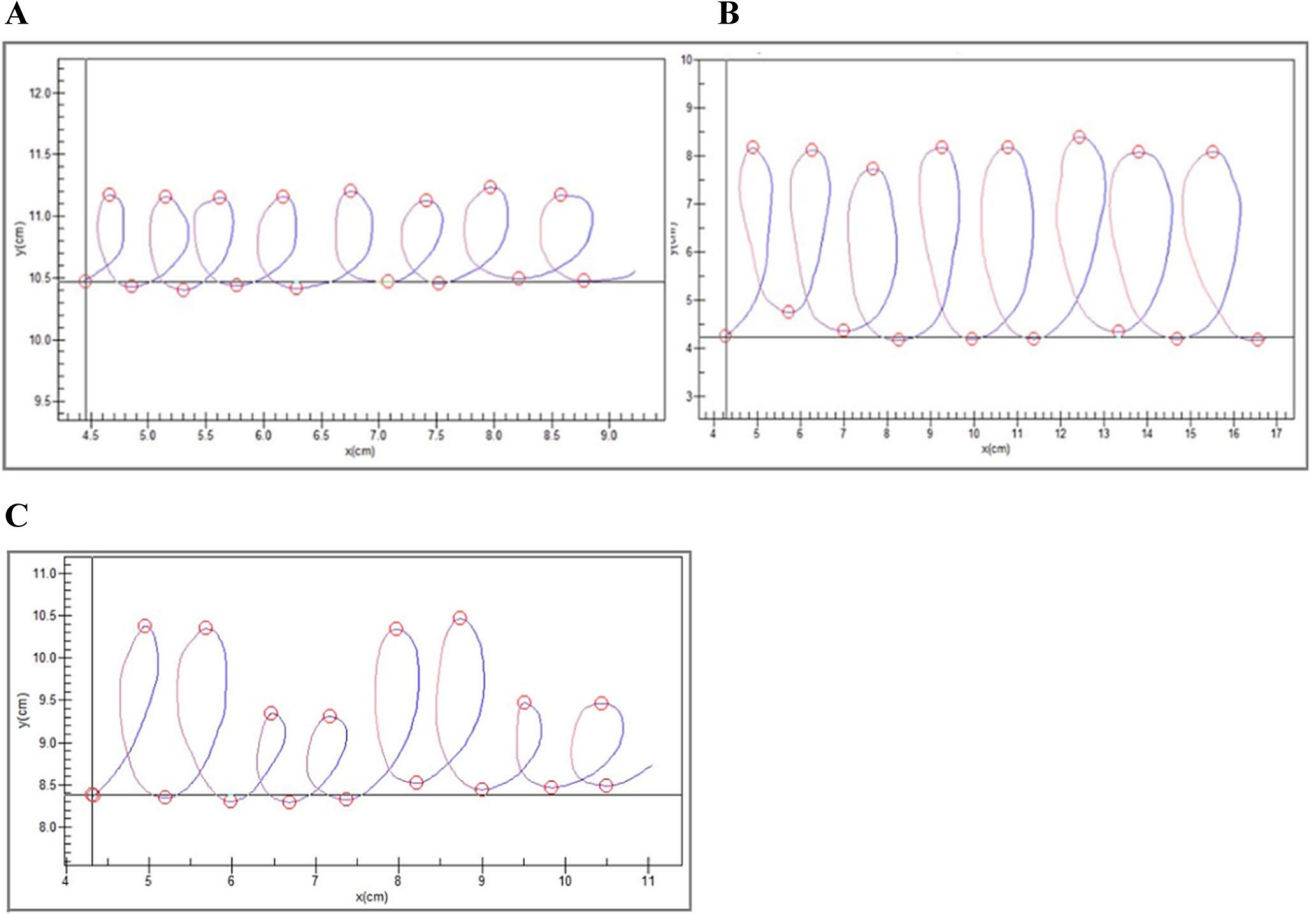
Example Handwriting Task trials and segmentation of (**A**) LL1, (**B**) LL4, and (**C**) LLee. Segmentation of the handwriting loops is depicted by the red circles (start and end of a segment) and color (blue = upward pen movement, red = downward pen movement). A. LL Velocity Scaling Score (HC vs. Lifetime MDD). B. LL4 Trial and Segmentation. C. LLee Trial and Segmentation

During these conditions, participants were instructed to write 8 loops continuously within either a 1 cm (LL1) or 4 cm (LL4) vertical boundary, using their dominant hand (note: all participants were right-handed) at their normal speed. The 8 loops from each trial were segmented into 16 vertical strokes (8 up, 8 down; see Fig. 1A and B) and processed for target variables separately for each condition: Peak Vertical Velocity and Vertical Size of stroke. Participants did three trials each of LL1 and LL4. Each trial lasted approximately 15 s. Valid trials included at least 10 segments (i.e., strokes).

#### LLee Condition

The LL (LL1 and LL4) conditions that typically measure VS (described above) are drawn separately (i.e., as 3 trials of LL1 loops and 3 trials of LL4 loops). We sought to explore whether whether VS derived from lines drawn within the same condition (i.e., alternative long and short lines from the LLee condition), may be more efficient probe of VS. During the LLee condition, participants were instructed to write alternating pairs of large and small loops (i.e., cursive “LLeeLLee”), using their dominant hand at their normal speed. The 8 loops from each trial were segmented into 16 vertical strokes (8 up, 8 down; see Fig. 1C). Valid trials included at least 10 segments (i.e., strokes). Participants did three trials of LLee.^1^ Each trial lasted approximately 15 s.

To examine the VS measure from the LLee condition, “LL” and “ee” segments were processed separately to calculate Peak Vertical Velocity and Vertical Size for each segment type. Of the 231 participants with Handwriting Task data, 6 participants were excluded for missing or too few LLee segments (final LLee *n* = 225).

### Analytical Approach

For handwriting task preprocessing details, see the Supplement. Regression analyses were conducted in R using the lme4 package (Bates et al., 2015). First, a slope variable was calculated for each participant, separately for the LL1/LL4 and LLee conditions. Peak Vertical Velocity (all segments) were regressed on Condition (dummy coded with the smaller condition set as the reference; LL: 1 cm = 0 and 4 cm = 1; LLee: 0 = ee, 1 = LL) with Vertical Size included as a covariate to account for individual variation in handwriting (Dean & Mittal, 2015). The slope coefficient for Condition was then carried forward into regression analyses. Steeper (positive) slopes reflect greater VS (and thus smaller PmR) from the smaller to larger segments: LL 1 to 4 cm and LLee “ee” to the “LL” segments.

For the first aim, a series of linear regression analyses were used to assess the following effects for Peak Velocity and VS: (1) 3-level Clinical Group (HC, rMDD, cMDD), dummy-coded with HC as the reference, (2) Anhedonia, and (3) General Depression. Effects for Vertical Size were also assessed to clarify whether effects were specific to velocity/VS or to other handwriting metrics. A planned follow-up analysis included Anhedonia and General Depression in a multiple regression model to identify whether either scale was uniquely related to VS. Overall *F*-values are reported for the Groups effects and standardized betas (β) are reported for the symptom effects.

The second aim was to examine the reliability of LL and LLee handwriting metrics from the conditions. The internal consistency of the handwriting metrics from the LL 1 cm, LL 4 cm, LLee “ee”, and LLee “LL”, conditions were examined by incrementally computing Cronbach’s alphas (Moran et al., 2013) using participants’ Peak Vertical Velocity. Cronbach’s alphas were initially computed for 2 segments (equal to 1 loop), and the number of segments included in the reliability analysis increased by 2 segments up to 48 segments (equal to 24 loops).

## Results

### Sample Demographics and Clinical Characteristics

As shown in Table 1, across groups participants reported primarily female sex and female gender, having non-Hispanic ethnicity, and varying races.^2^ Mean age was 29.3 years old (SD = 9.7).^3^ There were no significant group differences in the total number of segments that were retained across the LL, *F*(2,228) = 2.68, *p* =.071, or LLee conditions, *F*(2,222) = 0.50, *p* =.607.

### Handwriting Task and Depression

#### LL (LL1 Versus LL4)

**Fig. 2:**
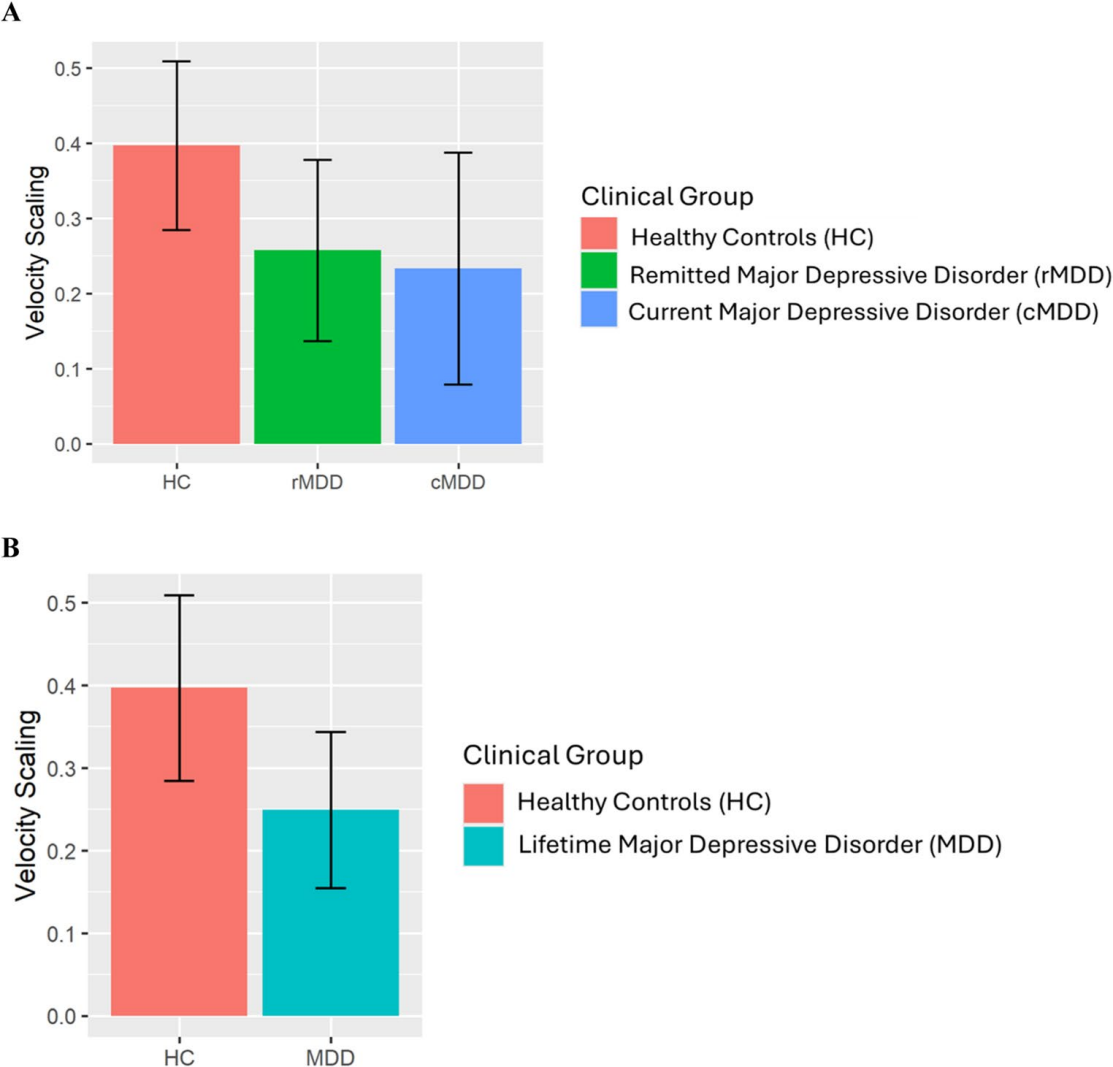
Depiction of the non-significant effect of Clinical Group on Velocity Scaling for the LL condition (LL1 vs. LL4). Clinical Group was tested using two approaches: (**A**) a 3-group comparison (HC, rMDD, and cMDD) and (**B**) a 2-group comparison (HC and lifetime MDD). Error bars represent the 95% CI. A. LL Velocity Scaling Score. B. LL Velocity Scaling Score (HC vs. Lifetime MDD)

Diagnostic groups did not significantly differ on Peak Vertical Velocity for the 1 cm, *F*(2,228) = 0.25, *p* =.778, or 4 cm condition, *F*(2,228) = 0.15, *p* =.863. As shown in Fig. 2A, LL VS did not significantly differ between the groups, *F*(2,228) = 1.90, *p* =.151. Results were comparable when HCs were contrasted with lifetime MDD (current and remitted MDD) for Average Peak Vertical Velocity for the 1 cm, *F*(1,229) = 0.34, *p* =.558, and 4 cm condition, *F*(1,229) = 0.29, *p* =.592. As shown in Fig. 2B, there was also no significant difference in LL VS between HCs and lifetime MDD (with rMDD and cMDD groups combined, although the group difference was trending, *F*(1,229) = 3.76, *p* =.054).

**Fig. 3:**
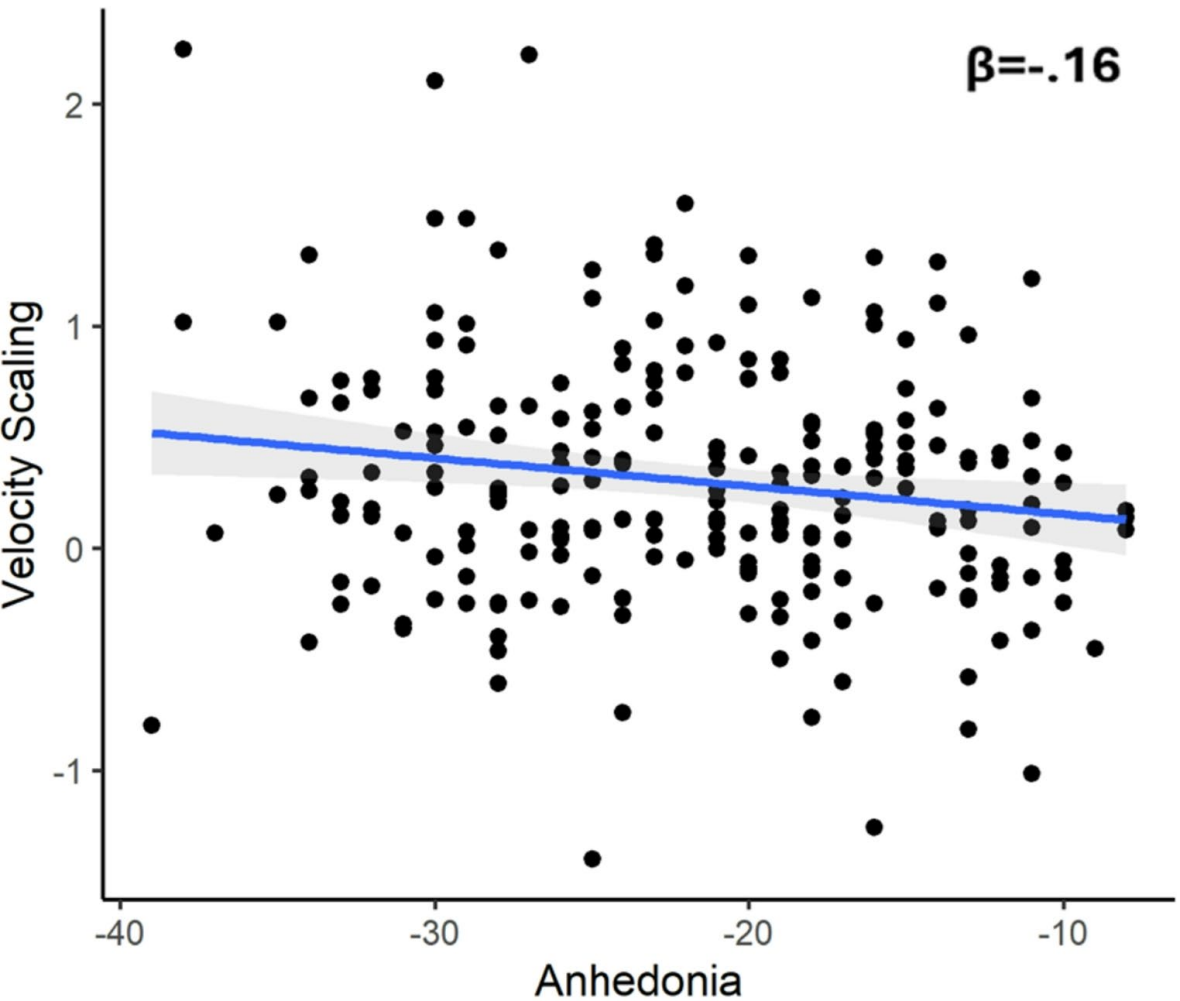
Figure depicting the signficiant association between Anhedonia (IDAS Well-Being scores multiplied by −1) and Velocity Scaling (slopes z-scored across all participants) for the LL condition (LL1 vs. LL4) across all participants (*p* <.05). Error bar represents the 95% CI and the standardized beta value is provided

Across all participants, LL VS was significantly related to Anhedonia, β=−0.16, *p* =.024. As shown in Fig. 3, higher symptoms of anhedonia were related to lower LL Velocity Scaling. By contrast, Anhedonia was not significantly related to Peak Vertical Velocity for the 1 cm condition, β =−0.03, *p* =.714, or 4 cm condition, β =−0.05, *p* =.492, highlighting the specificity of the effects to VS.

General Depression was not significantly related to Peak Vertical Velocity for the 1 cm, β = 0.08, *p* =.244, or 4 cm condition, β = 0.04, *p* =.614. LL VS was not significantly related to General Depression, β=−0.09, *p* =.155. When Anhedonia and General Depression were included as predictors in the same model, the effect of Anhedonia remained significant, β=−0.16, *p* =.048. No significant effects emerged for Vertical (see Supplemental Table S1).

####  LLee (LL Versus ee)

Groups did not significantly differ on Peak Vertical Velocity for either the ee, *F*(2,222) = 0.43, *p* =.652, or LL segments, *F*(2,222) = 0.03, *p* =.969. When HCs were contrasted with lifetime MDD, the results were comparable for Peak Vertical Velocity for the ee, *F*(1,223) = 0.59, *p* =.441, and LL segments, *F*(1,223) = 0.04, *p* =.847. LLee VS also did not significantly differ between the groups, *F*(2,222) = 1.18, *p* =.310 (see Supplemental Figure S1A). A follow-up analysis revealed that there was also no difference between HCs and lifetime MDD, *F*(1,223) = 0.31, *p* =.577 (see Supplemental Figure S1B).

Peak Vertical Velocity was not significantly related to Anhedonia for the ee, β =−0.01, *p* =.934, or LL segments, β =−0.01, *p* =.882. Finally, Anhedonia was not significantly related to Velocity Scaling, β =−0.03, *p* =.644 (see Supplemental Figure S2A).

General Depression was also not significantly related to Peak Vertical Velocity for the ee, β = 0.05, *p* =.461, or LL segments, β = 0.02, *p* =.737. Finally, General Depression was not significantly related to LLee VS, β =−0.06, *p* =.403 (see Supplemental Figure S2B). No significant effects emerged for Vertical (see Supplemental Table S1).

### Internal Consistency of LL1, LL4, and LLee

#### LL1 and LL4

**Fig. 4:**
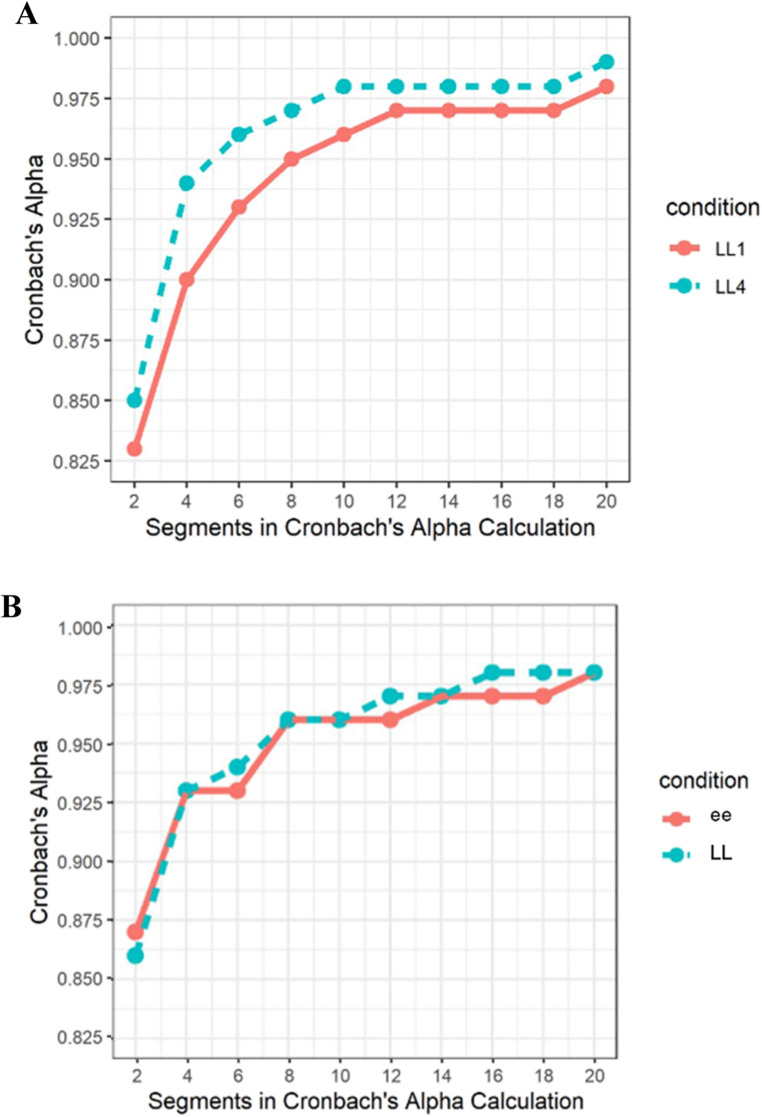
Internal consistency (Cronbach’s alpha) of the Handwriting Task is depicted using an increasing number of segments included in the reliability calculation for the (**A**) LL and (**B**) LLee conditions. For each condition, the consistency of Peak Vertical Velocity across handwriting segments was calculated starting with 2 segments and was re-calculated with an increasing number of segments (in increments of 2) up to 48 segments. Cronbach’s alphas were calculated separately for LL1, LL4, ee, and LL A. LL Internal Consistency. B. LLee Internal Consistency

**Fig. 5:**
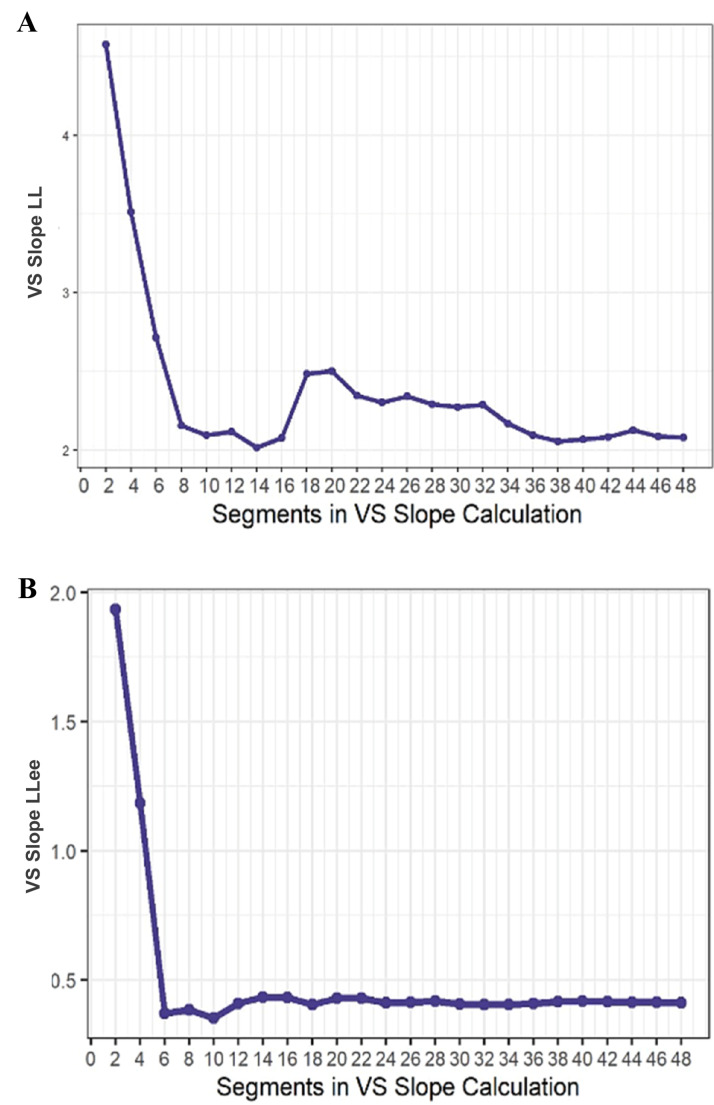
Stability of Velocity Scaling (VS) based on the number of segments that are included in the VS calculation for the (**A**) LL and (**B**) LLee conditions A. LL Slope Stability. B. LLee Slope Stability

LL1 and LL4 Peak Vertical Velocity exhibited excellent internal consistency, even at only 2 loops (i.e., 4 segments; 1 cm Cronbach’s α = 0.95 and 4 cm Cronbach’s α = 0.97; see Fig. 4A). The LL VS slope began to stabilize around 4 loops (i.e., 8 segments), but increased around 9 loops (i.e., 18 segments, approximately the end of trial 1 and the start of trial 2). The VS slope restabilized around 18 to 20 loops (approximately during trial 3; see Fig. 5A).

#### LLee

LLee “ee” and LLee “LL” Peak Vertical Velocity also exhibited excellent internal consistency, even at only 2 loops (ee Cronbach’s α = 0.93 and ll Cronbach’s α = 0.93; see Fig. 4B). The LLee VS slope began to stabilize around 3 loops (see Fig. 5B).

## Discussion

The main goals of the present study were to (1) examine associations between a handwriting task that assessed VS - a behavioral indicator of PmR, and MDD diagnosis and dimensional measures of depression and (2) assess the reliability of the handwriting task via internal consistency. We also assessed these psychometric properties for velocity, which is related to VS, but is not specific to difficulties with scaling rate of movement.

The first goal of the present study was to assess relations between velocity, VS, and depression. Although MDD was previously found to be related to lower velocity (wrist flexion, Caligiuri & Ellwanger, 2000; handwriting, Sabbe et al., 1999) and VS (Caligiuri & Ellwanger, 2000; Lohr et al., 2013), in the present study none of the handwriting metrics differed based on categorical MDD (either current or lifetime). Our results are in line with at least one other study that did not find a group effect of MDD for VS based on wrist flexion (Caligiuri et al., 2003). This suggests that the lack of a significant group effect may not be entirely attributable to the use of a handwriting measure (discussed further in the paragraph below).

Despite not finding a significant effect of MDD diagnosis, there was a significant relationship found between anhedonia and VS. Specifically, across all participants, greater anhedonia was related to lower VS. This is in line with previous research finding (1) relations between anhedonia and PmR in individuals with depression (Lemke at al., 1999) and Parkinson’s Disease (Lemke et al., 2005), and (2) inverse associations between handwriting-based VS and negative symptoms of schizophrenia, which include anhedonia and avolition (Dean & Mittal, 2015). Importantly, the relation between anhedonia and VS held above and beyond general depressive symptoms, highlighting the specificity of the effects to anhedonia.

Although speculative, there are several reasons why anhedonia may be related to poorer VS. First, it is plausible that impaired VS ability may contribute to anhedonia through its impact on motor-dependent goals and outcomes. For example, difficulty scaling the rate of task speed could result in difficulties completing work in a timely manner or failure to meet deadlines (Barch et al., 2014), which may contribute to a sense of helplessness, loss of motivation, and loss of interest. Alternatively, anhedonia may contribute to poorer VS through its effects on motivation, drive, and persistence, which could result in reduced motor initiation and less sustained motoric activity (Lemke et al., 1999). Finally, there may be a common underlying process that affects both VS and anhedonia. For example, disrupted basal ganglia function, atypical dopaminergic signaling, and/or altered motor and subcortical circuitry (e.g., the corticostriatal-thalamocortical circuit) may contribute to both slower VS and anhedonia (Bologna et al., 2020; Chung & Barch, 2015; Cortese et al., 2005; Zhang et al., 2016).

More broadly, the different findings for the categorical diagnosis of MDD and dimensional symptoms may, in part, speak to the importance of considering the significant symptom heterogeneity within MDD. Indeed, there are over 1,000 different combinations of symptoms that can lead to an MDD diagnosis (Fried & Cramer, 2017; Fried & Nesse, 2015). Relative differences in symptoms across MDD samples (e.g., less anhedonia, less motor impairment, and/or greater sadness) may impact whether group differences in velocity and VS emerge.

The second goal of the present study was to assess the psychometric properties of a handwriting-based instrumental measure of PmR. Velocity exhibited excellent internal consistency for all conditions. Notably, internal consistency reached a level that was excellent (≥ 0.90) for both LL and LLee conditions with only 2 loops (4 segments) and reached ≥ 0.97 by 10 loops (20 segments). Additionally, VS scores (LL and LLee) all stabilized before the end of the third trial of loops, indicating that 3 trials of 8 loops is adequate for achieving excellent reliability and VS slope stability. Overall, the results extend prior preliminary internal consistency results for velocity and VS in individuals with schizophrenia (Caliguri et al., 2010, which assessed internal consistency at the trial level) by providing further evidence that a handwriting task delivered using a digital tablet, can reliably detect motor slowing.

This study also tested the VS in a novel way, where both small and large segments were made within the same condition (i.e., LLee). We found that the velocity for the smaller “ee” and larger “LL” both exhibited excellent internal consistency. However, the VS measure from this condition was not significantly related to any measure of depression - MDD, anhedonia, or general depressive symptoms. One reason for the poorer validity for the VS measure for LLee is that unlike in the LL condition, participants must quickly shift between making “LL” and “ee” loops during the LLee condition, potentially resulting in a less precise VS measure (i.e., LLee VS scores reflect not only VS ability, but also within condition set-shifting). Taken together, this study suggests that examining the scaling between small and large loops yields a more valid measure of psychomotor slowing if done in separate conditions rather than within the same condition.

There are several strengths of the present study that should be noted. First, the reliability and stability of the handwriting task metrics were computed at the segment level (as opposed to the trial level; e.g., Caligiuri et al., 2010), which included 16 segments for each trial (i.e., 8 loops). This allowed for a more fine-grained analysis of reliability and stability than in previous studies. Critically, this revealed that whereas the internal consistency of velocity reached a high level with very few segments, LL VS did not stabilize until around 36 segments (i.e., within the third trial of loops). Second, the reliability and validity of the handwriting task were assessed in relation to MDD using both a categorical (HC, cMDD, rMDD) and dimensional approach (anhedonia and general depressive symptoms). This allowed for an examination of whether MDD and/or specific dimensions of MDD (which is a very heterogenous disorder) were related to impaired motor functioning. Finally, the study included a relatively large and racially and ethnically diverse sample, thereby supporting the generalizability of the results to a broader population.

There are also several limitations that should be noted. First, although there was a significant relation between anhedonia and VS, we do not know the direction of this relationship. Whereas impaired VS may contribute to anhedonia, the alternative is also plausible. Second, this study excluded participants who were left-handed due to the association between neural lateralization and handedness. Left-handedness is unlikely to affect VS and handwriting task reliability systematically, but should be considered in future studies, particularly if this task is used in clinical settings, since approximately 10% of the Western population is left-handed (Mariani et al., 2023). Finally, VS scores were measured at only one time point and may be affected by state-dependent factors (such as fatigue), as well as factors that are not specific to depression-related PmR (general difficulties with motor functioning/coordination). Furture studies should explicity account for these potential confounds, as well as examine the overlap between (depression-related) clinical measures of PmR and VS.

In sum, the present study found a significant and robust association between VS and anhedonia symptoms. Given the handwriting tasks’ high internal consistency and feasibility, it should be considered as a potential indictaor of PmR in clinical settings. Additionally, the digital handwriting task was found to reliably (consistently) evaluate velocity and VS. The task does not require extensive training and can be completed within only a few minutes. Furthermore, as previously noted, the measure can quantify fine-grained motor symptoms (e.g., difficulties scaling movement) that are not readily captured by self- and observer-report.

Future studies are needed to determine the temporal course of VS (e.g., how long it exhibits stability over time and for whom), the temporal relationship between impaired VS and anhedonia, and whether VS indexes vulnerability to future depression. If VS scores provide a meaningful index of future risk for MDD and/or specific MDD symptoms, this would provide further support for the clinical utility of the handwriting task. Given that other instrumental measures that measure PmR may also be clinically useful (i.e., an eletrogoniometer to measure wrist flexion), future studies could also consider directly comparing the handwriting task metrics that were assessed in the present study with other handwriting metrics (Hegerl et al., 2005; Tucha et al., 2002) and other behavioral measures of VS (e.g., wrist flexion, Caligiuri et al., 1998). Additionally, to identify relative costs and benefits of available assessments, these measures should be compared with comprehensive clinical evaluations of PmR.

## Supplementary Information

Below is the link to the electronic supplementary material.

ESM 1 (DOCX 295 KB)

## Data Availability

The data from the experiments reported here are available in the NIH National Data Archive.

## Footnotes

1 Note: “LLee” is referred to as the CO2 condition by the MovAlyzeR software.

2 By design, female control participants were over-recruited at a higher rate than males so that the groups would be matched on gender, given the higher prevalence of MDD among females.

3 VS was not significantly related to Sex, *F*(1,229) = 1.48, *p* =.225, Gender, *F*(1,227) = 1.04, *p* =.355, Age, *F*(1,229) = 0.74, *p* =.390, and Medication Status *F*(1,229) = 2.12, *p* =.147. Including covariates in the analyses did not result in any meaningful changes to the results (e.g., no significant results became non-significant).
