# Supplementary material for: Anhedonia Relates to Increased Psychomotor Retardation Using an Instrumental Handwriting-Based Measure
Source: J Psychopathol Behav Assess. 2025 Sep 22;47(4):74. doi: 10.1007/s10862-025-10249-1 (PMC12454474; doi:10.1007/s10862-025-10249-1)
Supplement: Supplementary file 1 — (DOCX 295 KB) [file 10862_2025_10249_MOESM1_ESM.docx]

Anhedonia Relates to Increased Psychomotor Retardation Using an Instrumental Handwriting-Based Measure

**Supplementary Material**

**Handwriting Task Preprocessing Steps**

Handwriting data was processed using a semi-automated procedure. First, automated procedures were applied via the MovAlyzeR software. Following an approach used by Dean et al. (2013) and Caligiuri et al. (2019), trials were processed using a Fast Fourier Transform (FFT) Low Pass 12 Hz filter, and initial downstroke and trailing pen lifts were removed. Second, a locally developed R script was used to filter out initial downstroke and trailing pen lifts that were not removed by the MovAlyzeR software and flag trials for visual inspection (e.g., all trials that included more than 16 segments). Additional segments (i.e., more than 16 per trial) were retained when the segments were part of a full loop, otherwise they were filtered out.

**Tables**

**S1. Relations Between Depression Measures and Vertical Size**

| **Condition** | | **Clinical Group** | | **IDAS-II Subscale** | |
| --- | --- | --- | --- | --- | --- |
|  |  | **HC vs. rMDD vs. cMDD** | **HC vs. Lifetime MDD** | **Anhedonia** | **General Depression** |
| **LL4/LL1** | 1 cm | *F*(2,228)=0.49, *p*=.614 | *F*(1,229)=0.01, *p*=.906 | β=-.05, *p*=.456 | β=-.05, *p*=.497 |
|  | 4 cm | *F*(2,228)=0.49, *p*=.615 | *F*(1,229)=0.29, *p*=.583 | β=-.13, *p*=.051 | β=-.01, p=.838 |
| **LLee** | LL | *F*(2,222)=0.99, *p*=.370 | *F*(2,223)=0.71, *p*=.399 | β =-.11, *p*=.090 | β =-.07, *p*=.292 |
|  | ee | *F*(2,222)=0.05, *p*=.948 | *F*(2,223)=0.02, *p*=.889 | β =-.07, *p*=.283 | β =-.01, p=.828 |

**Figures**

**S1A and S1B**

**A. LLee Velocity Scaling Score (HC vs. rMDD vs. cMDD)**


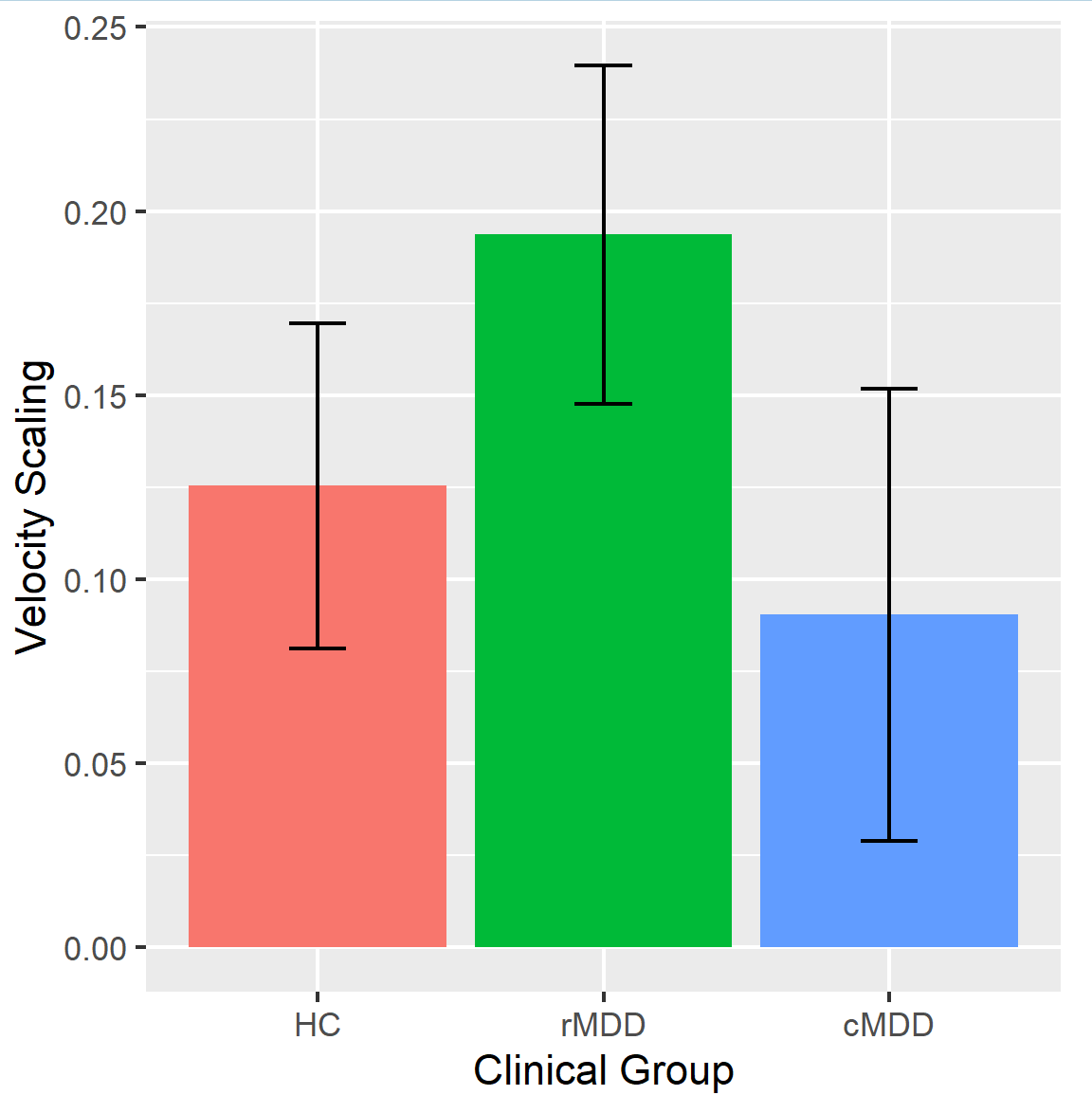


**B. LLee Velocity Scaling Score (HC vs. lifetime MDD)**


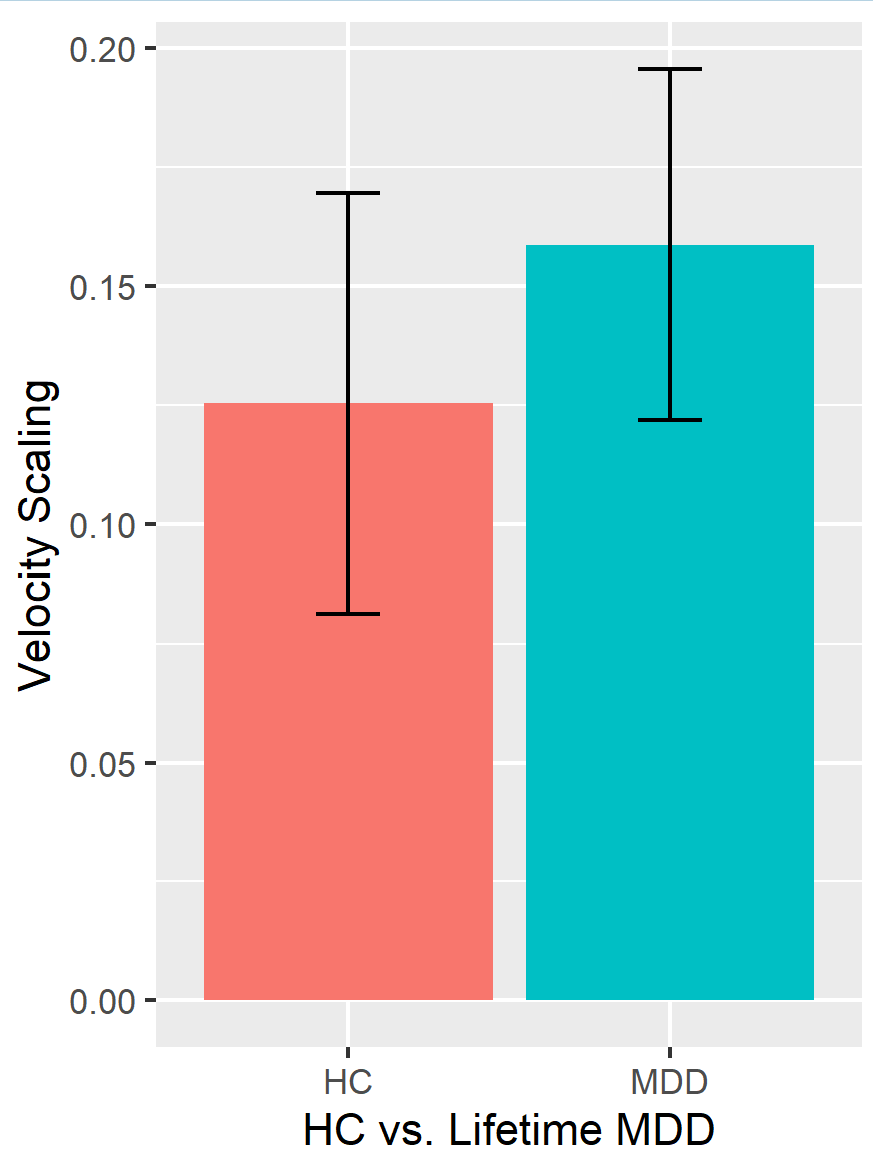


**S2A and S2B**

**A. Anhedonia (IDAS-II) and LLee Velocity Scaling Score**


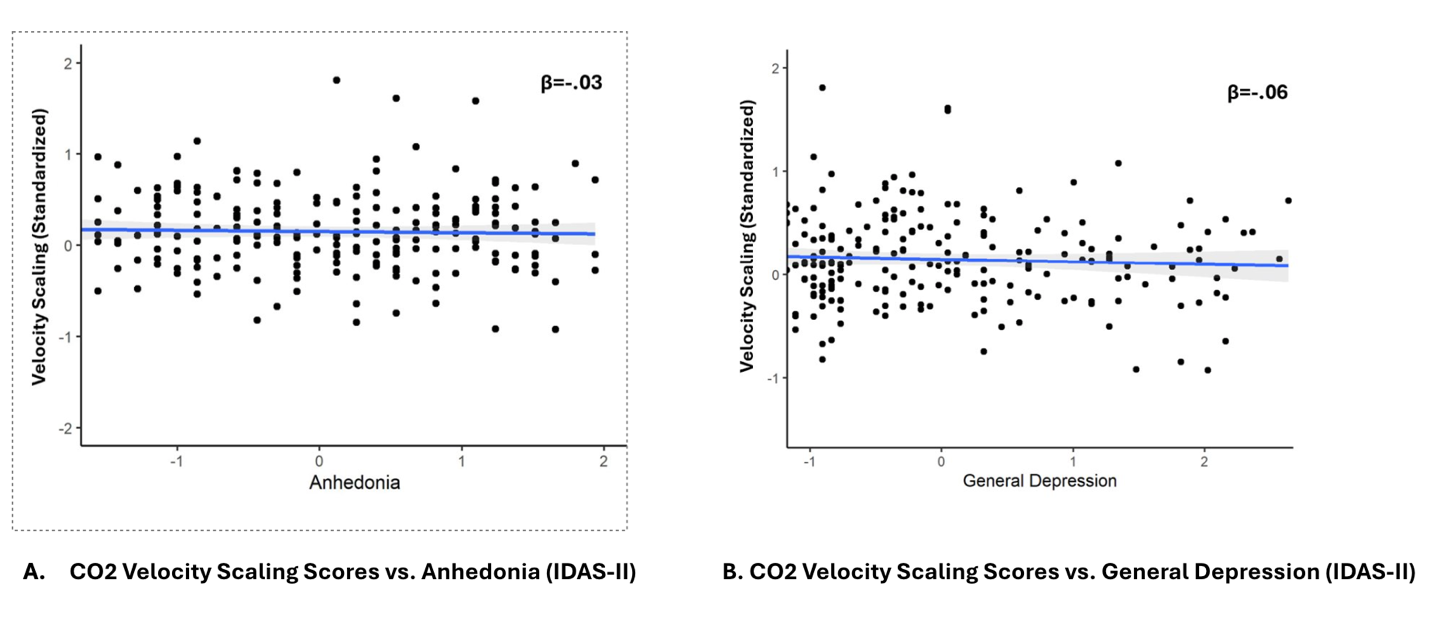


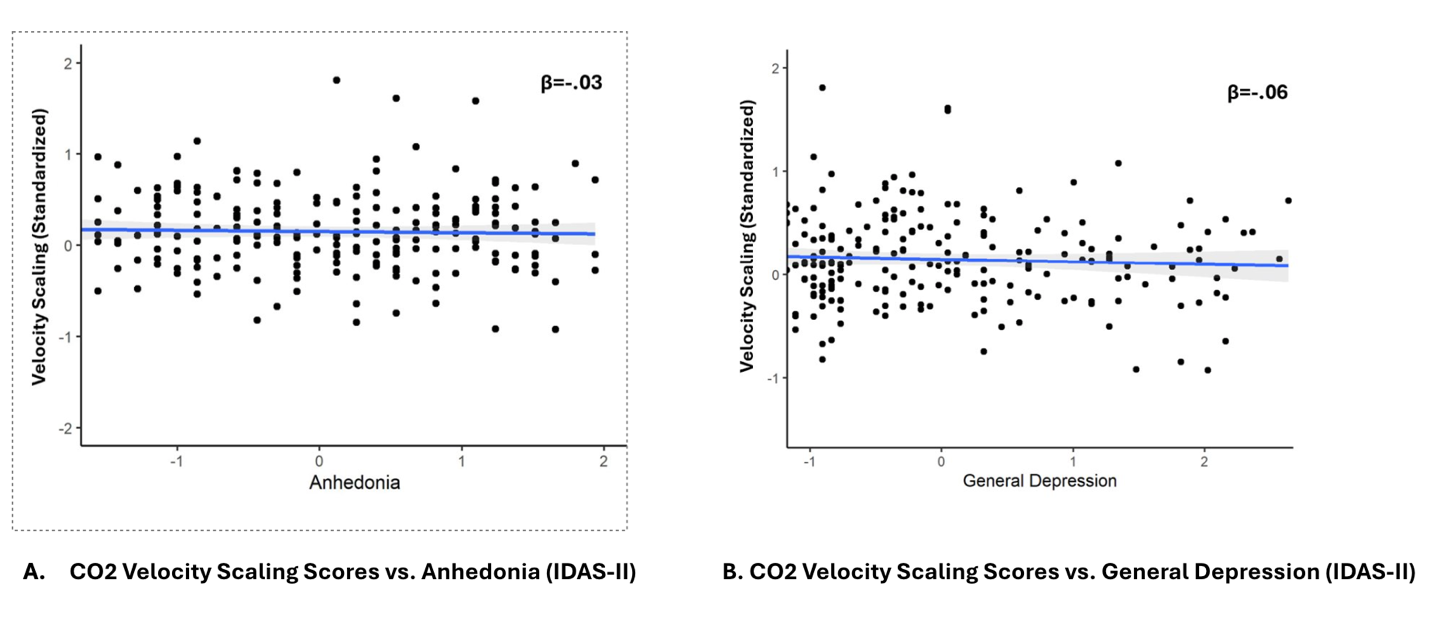
**B. General Depression (IDAS-II) and LLee Velocity Scaling Score**
